# Enhancing membrane-based soft materials with magnetic reconfiguration events

**DOI:** 10.1038/s41598-022-05501-7

**Published:** 2022-02-01

**Authors:** Michelle M. Makhoul-Mansour, Joyce B. El-Beyrouthy, Leidong Mao, Eric C. Freeman

**Affiliations:** 1 School of Environmental, Civil, Agricultural and Mechanical Engineering, University of Georgia, Athens, GA 30602 USA; 2 School of Electrical and Computer Engineering, University of Georgia, Athens, GA 30602 USA

**Keywords:** Mechanical engineering, Bioinspired materials, Self-assembly

## Abstract

Adaptive and bioinspired droplet-based materials are built using the droplet interface bilayer (DIB) technique, assembling networks of lipid membranes through adhered microdroplets. The properties of these lipid membranes are linked to the properties of the droplets forming the interface. Consequently, rearranging the relative positions of the droplets within the network will also alter the properties of the lipid membranes formed between them, modifying the transmembrane exchanges between neighboring compartments. In this work, we achieved this through the use of magnetic fluids or ferrofluids selectively dispersed within the droplet-phase of DIB structures. First, the ferrofluid DIB properties are optimized for reconfiguration using a coupled experimental-computational approach, exploring the ideal parameters for droplet manipulation through magnetic fields. Next, these findings are applied towards larger, magnetically-heterogeneous collections of DIBs to investigate magnetically-driven reconfiguration events. Activating electromagnets bordering the DIB networks generates rearrangement events by separating and reforming the interfacial membranes bordering the dispersed magnetic compartments. These findings enable the production of dynamic droplet networks capable of modifying their underlying membranous architecture through magnetic forces.

## Introduction

Materials inspired by cellular organisms approximate selected characteristics of living cells^1,2^ and are used in applications such as drug delivery^3^, biocompatible sensors, and novel soft robotic actuators^4,5^. One of these criteria, adaptability, or the ability to change functionality or form when needed, is crucial for the development of autonomous bioinspired materials. In this manuscript we will primarily focus on implementing a form of structural adaptability involving shape-shifting capabilities in bioinspired materials. Inspired by how cellular tissues structurally adapt, we will explore how magnetically driven shape shifting capabilities can be implemented in materials constructed using the droplet interface bilayer (DIB) technique, a popular technique for assembling lipid membranes that may be used to recreate cellular phenomena^6^. Building on previous works^7,8^, ferrofluids will be integrated into our DIB-systems and used to reconfigure neighboring lipid membranes through magnetic forces.

The DIB technique^6,9^ assembles phospholipid bilayers at the interfaces of lipid-coated aqueous droplets dispersed in an oil environment^10,11^, enabling the creation of soft bioinspired structures^6,12,13^. Phospholipids may be dispersed in either phase or in both phases simultaneously^14–17^. Upon bringing the lipid-coated droplets into contact, a lipid bilayer forms spontaneously in a zipping mechanism expelling the oil and adhering the droplets together^10,11,18^. Notably, the DIB technique may be used for the construction of membrane-based materials^15,19–24^ as its liquid-in-liquid geometry allows for the sequential assembly of multiple lipid bilayers in complex networks with collective functionalities^12,13,19,25–27^.

DIB tissues often retain their original structure over the course of the experiment. These cases permit for the study of membrane biophysics or the mechanics of interfaces and self-assembly, yet the capacity of the DIB networks for adaptation in response to external stimuli is limited. To rectify this limitation, recent efforts have proposed that DIB-based materials can be functionalized to adapt to external triggers by either (a) changing their internal chemical composition or (b) shape-shifting^6^. The former (composition evolution) is traditionally accomplished via targeted permeabilization of the internal interfacial bilayers allowing for molecular between droplets flow akin to chemical computing^28,29^. Interfacial bilayers are often functionalized either with stimuli-responsive molecules (enabling sensitivity to light^26,30^, voltage^31,32^, or mechanical inputs^33–35^) or by impacting the packing of lipid molecules^17,36,37^. Meanwhile, the latter (shape-shifting) is accomplished by creating changes in the layout of aqueous compartments in response to suitable environmental triggers. This work develops the latter case, investigating shape-shifting capabilities within DIB-structures that follow mechanisms inspired by natural tissues using magnetic forces as a trigger.

Recent research efforts have also explored manipulating and tuning the structure of the DIB membranes. In single DIB pairs, the dimensions of the adhered interfaces are often modified either using direct mechanical contact^33,34,38,39^ or electrowetting^40–43^. However larger collections of droplets produce additional complexities for these approaches. As an alternative, morphing DIB-based structures have been generated either using combinations of thermally-responsive hydrogels^20^ and light-responsive nanoparticles^44^ or osmotic swelling^19^. Furthermore, optics-based manipulation have been investigated using optical tweezers^45,46^ or magnetic particles^7,8,15,44^ for either controlling translational motion of the droplet networks or for the initial assembly of the interfaces. Each of these approaches for dynamic DIB materials primarily focuses on changes in the overall network shape rather than the organization of the individual droplets within the network.

**Figure 1:**
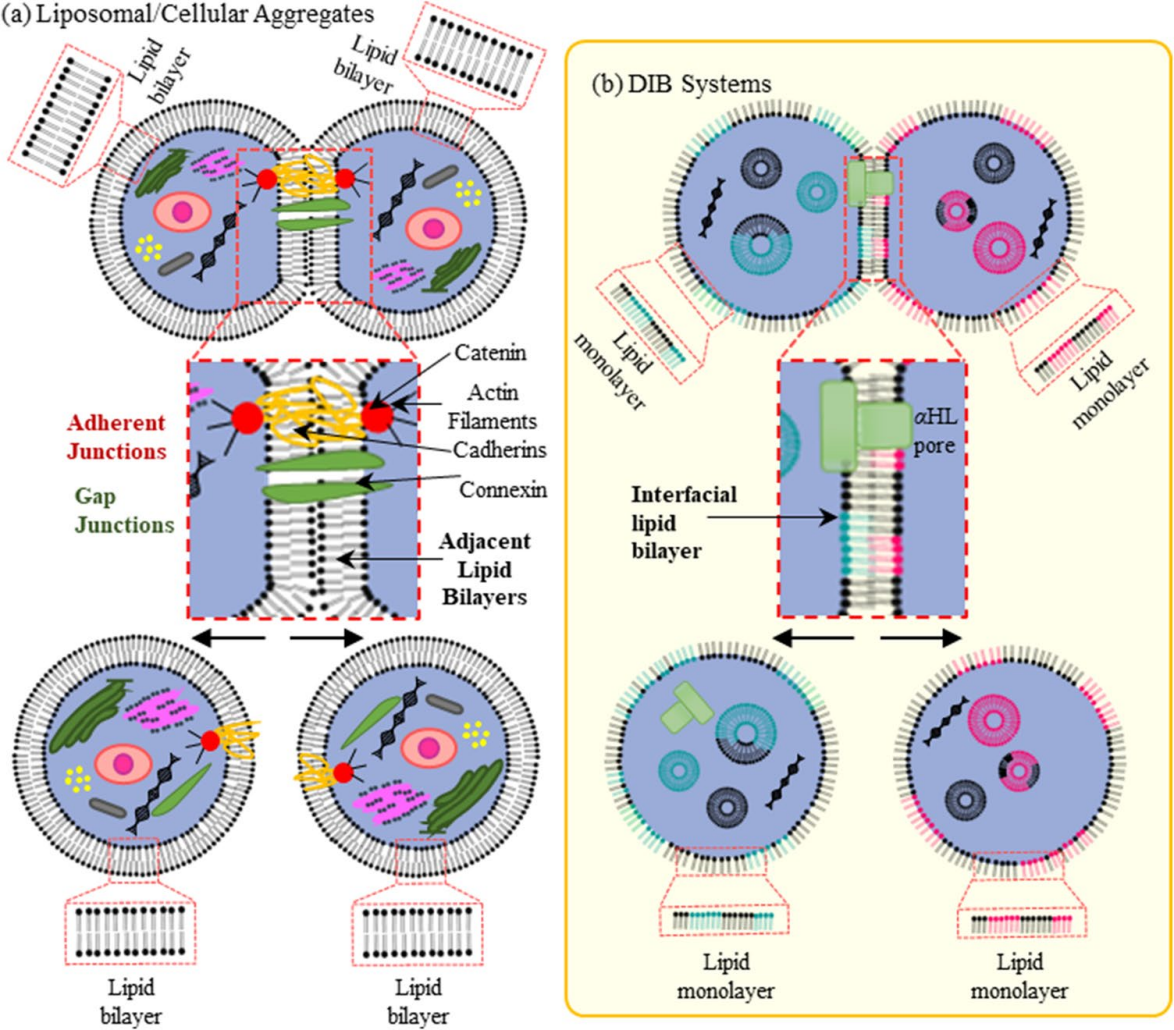
Schematic representation of adhesion in (**a**) cellular tissues and (**b**) DIB assemblies. According to the differential adhesion hypothesis (DAH), tissues may be described as emulsive systems which reorganize spontaneously to minimize their interfacial free energy, similar to adhered droplets in emulsions. In natural living tissues and liposomal aggregates, interfaces are comprised of two adjacent lipid membranes. In contrast, DIB-systems form interfaces between adhered lipid monolayers producing a single lipid membrane. This schematic representation was created by the authors using Microsoft PowerPoint.

The unique contribution of this research involves the rearrangement of the individual droplets within the network, breaking the droplets apart then forming new membranes. This takes advantage of the metastable nature of the DIB networks, using magnetic forces to shift the droplets between different adhered arrangements. Combining a reconfigurable DIB network with embedded transporters permits adjustable patterns of internal exchange, especially when combined with transmembrane exchanges dependent on lipid membrane asymmetry^17,36,37,47,48^ as the old membranes separate and new ones are formed (Fig. 1b).

This approach is inspired by living tissues. Cellular tissues modify their orientation, shape, density and overall structure through rearrangement events of the underlying cells, granting an intercellular stress profile^49,50^ best optimized for their environment. These structural changes in living tissues are partially accomplished by spatially tuning the interfacial tensions at the individual interfaces^51–54^ (Fig. 1a). These cell–cell and cell-medium tensions are analogous to the bilayer and monolayer tensions in DIBs respectively. However, in the droplet-based tissue, this tension is largely determined by the selected solvent and lipid compositions which present challenges for adjusting individual bilayer tensions in DIB tissues^16,18,41,55^. Instead, an alternative method for generating forces that targets only selected droplets within the tissue is necessary. In this research we propose the use of magnetic fields coupled with distributed ferrofluid droplets to generate localized forces in the DIB structures, driving droplet decoupling and rearrangement. Previous work has shown that aqueous ferrofluids may be safely encapsulated within single droplets within the DIB network, functionalizing select droplets with magnetic susceptibility^7^ and creating a magnetically heterogeneous DIB network.

First, we examine magnetic manipulation of a single DIB interface through a combination of modelling and experimental work. Next, we produce heterogeneous structures of magnetic and non-magnetic aqueous droplets within an oil medium, centered between four electromagnets. Magnetic fields are then activated to selectively apply forces within the collections of adhered droplets. The magnetic ferrofluid subcompartments within the structure collectively respond to the magnetic field and adjust accordingly, rearranging the adhered network between different metastable configurations. Finally, we show how this may be used to enable communicative pathways within the tissue using magnetic fields. The proposed reconfigurable DIBs are inspired by phenomena observed during morphogenesis^49,50,53,56^ including T1 events^56,57^, invagination^58^ and non-apoptotic extrusion^56^.

Our presented results are the first demonstration of dynamic reconfiguration within DIB tissues, where shape-change occurs through the separation and reformation of the lipid membranes. While magnetic manipulation has been achieved previously^15^ as well as shape-shifting through swelling and shrinking phenomena^13^, the emphasis here is on how ferrofluid droplets may be used to exert localized forces on their neighbors for the rearrangement of the internal droplet organization. Since the functionality of a single DIB interface is determined by the two comprising droplets, this allows for varying network functionality through reconfiguration. Notably this technique may be accomplished in an encapsulated system and is contact-free. Potential applications include plastic rearrangement events for neuromorphic materials^59^, controlled droplet–droplet exchanges^17^, and the continued development of dynamic functional droplet networks^6^.

## Results

### Manipulating single lipid interfaces using a magnetic field

**Figure 2:**
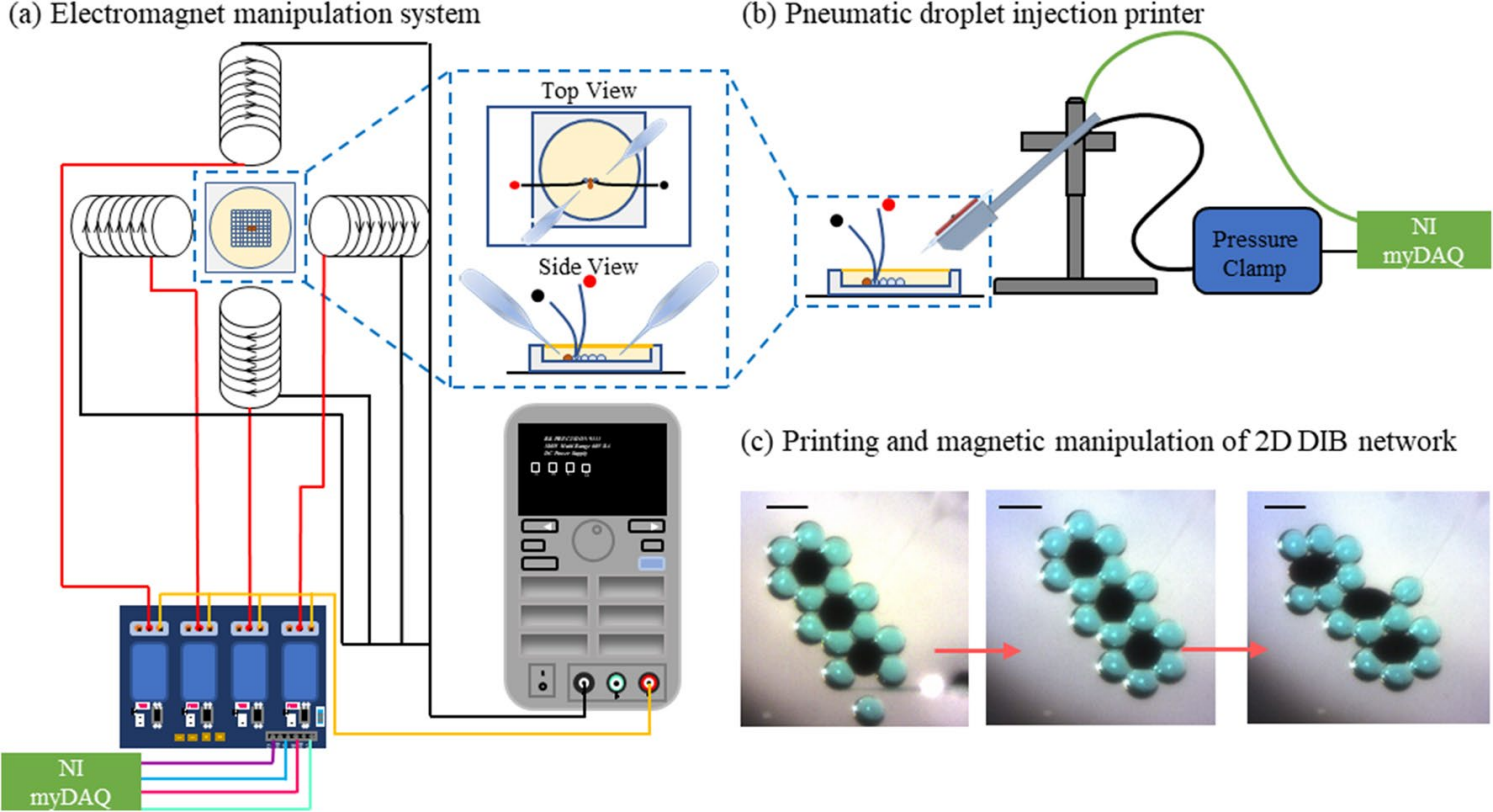
Schematic representation of the magnetic stage: the pneumatic microdroplet printer and the electromagnet array. (**a**) Four solenoids with paramagnetic cores (EFI alloy) are placed as shown and used to magnetically control the position of ferrofluid droplets injected into the oil medium. The power input of the solenoids is computer controlled with a user interface connected to an external microcontroller (ARDUINO), a relay system and a power supply. (**b**) Microdroplets are deposited into the oil dish using a computer-controlled pneumatic system. Microinjectors are pressurized using a pressure-clamp system connected to an external microcontroller (ARDUINO). These schematic representations were created by the authors using Microsoft PowerPoint. (**c**) Various compositions of DIB structures can be constructed by depositing magnetic (EMG 507—black color) and non-magnetic (aqueous buffer—blue color) microdroplets into an oil medium. Droplets are then brought into contact using a user-driven micropipette; interfacial bilayers are formed giving rise to a stable 2D membranous structure. Scale bars represent 600 µm each.

**Figure 3:**
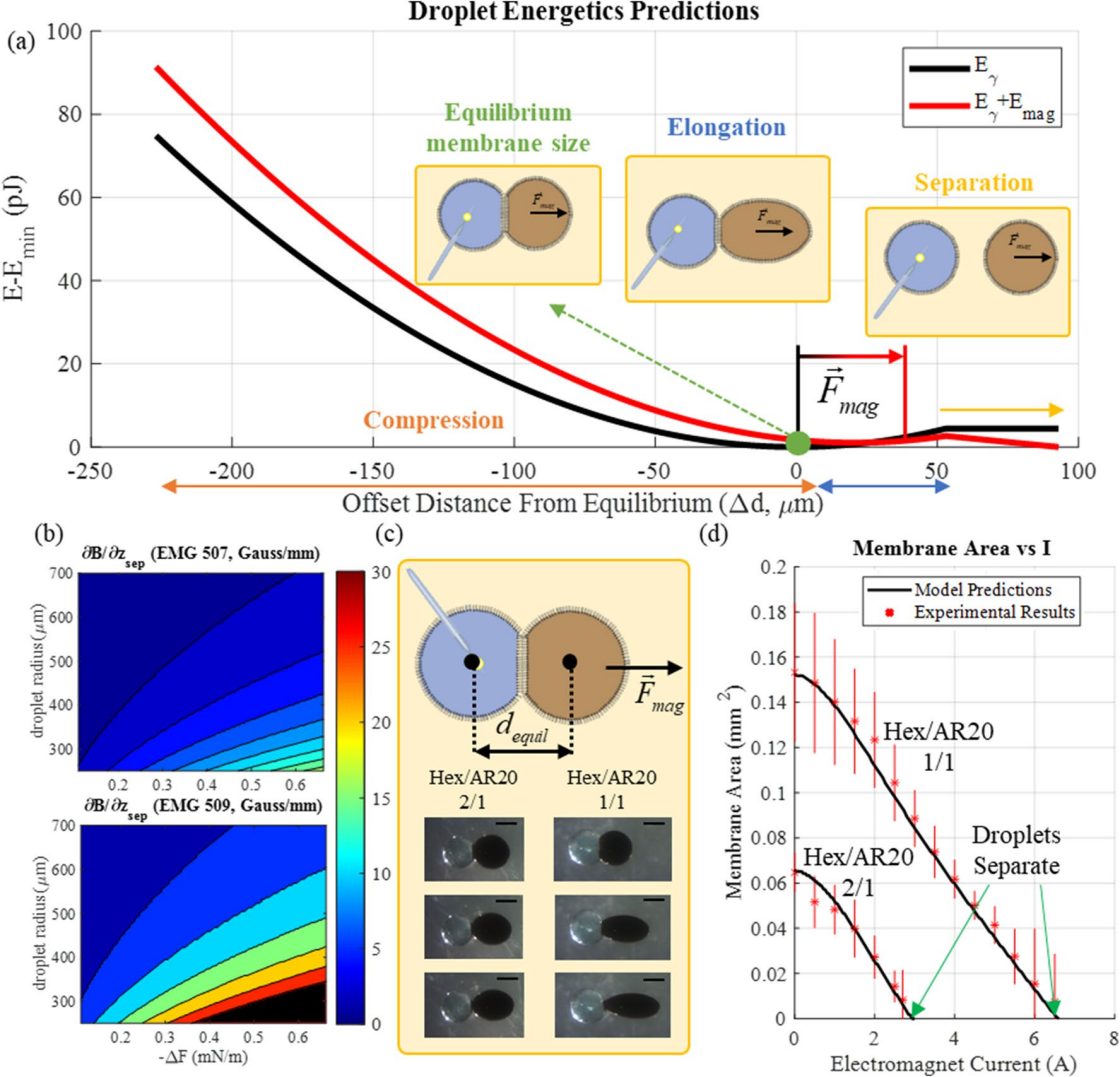
(**a**) Schematic representations (top view) of the manipulation of single membrane bordered by a ferrofluid droplet for different magnetic fields. An aqueous droplet is attached to an anchor. A ferrofluid droplet is then deposited in the same oil medium and a bilayer formed at the interface of these two droplets. Upon electromagnet activation, the ferrofluid droplet begins to pull away from the anchored droplet. This may be described by plotting the summed energy (magnetic and interfacial) and locating the minimum (Hexadecane/Silicone Oil AR20 2/1 volume mixture). (**b**) Necessary gradient of the magnetic field predicted for droplet separation as a function of the droplet radius and energy of adhesion for EMG509 and EMG507. Solvents that produce more stable bilayers resulting in higher energy of adhesions require more force to separate. Smaller droplets are more difficult to manipulate since the magnetic energy scales with volume and the interfacial energy scales with area. For lower concentration ferrofluids, this results in complete saturation prior to separation (shaded here in black). (**c**) Schematic representations and experimental images of membrane manipulation using electromagnets. A ferrofluid droplet (EMG507) is adhered to an anchored aqueous droplet and an electromagnet to the right is activated. The external oil phase is varied and the variation in droplet adhesion is hence explored. Scale bar represents 500 μm. (**d**) The current supplied to the electromagnet is varied and the average membrane area with respect to current is plotted and compared against model predictions for two different solvent compositions. Schematic representations in (**a**) and (**c**) were created by the authors using Microsoft PowerPoint.

First, we investigate the mechanics involved in manipulating a single membranous interface using the equipment presented in Fig. 2, printing DIB networks with dispersed ferrofluid compartments and manipulating them with a collection of electromagnets positioned around the working stage. For the initial experiments, a single ferrofluid droplet was adhered to an aqueous droplet anchored to a hydrogel-coated pipette and a magnetic field is supplied from the right as depicted in Fig. 3c. These hydrogel anchors are necessary for fixing one droplet in place as otherwise the two droplets will glide together across the surface of the dish in response to the magnetic field rather than separating. The current supplied to the electromagnet is gradually increased while the bilayer area between the two droplets is measured visually as the droplets separate, demonstrating magnetic control over bilayer dimensions. The droplets are allowed to reach equilibrium before each measurement.

An energetics model was used to illustrate how the experimental parameters will influence the rearrangement of the droplets with a magnetic field, and predictions are compared to experimental results and then used to select the optimal ferrofluid/oil combination. This energetics model combines both the interfacial energy of the DIB with the magnetic energy produced by the magnetization of the ferrofluid droplet in response to electromagnetic activation (more details discussed in the Experimental Methodology section). The interfacial energy (*E*_*γ*_) may be described by the monolayer and bilayer tensions (*γ*_*m*_, *γ*_*b*_) multiplied by their respective areas (*A*_*m*_, *A*_*b*_) as described in Eq. (1)^54,60^. The areas are approximated by assuming that the droplets will behave as adhered spherical caps with constant volumes. This assumption produces equations linking the distance between the droplet centres, their apparent radii, and the area of their adhered interface. Varying the distance between the droplet centres and solving numerically for the produced droplet geometry allows for a plot of the interfacial energy with respect to the distance between the droplets’ centres measured from equilibrium as shown in Fig. 3a,c. All inputs for these variables are provided in Supplementary Tables S1–S3 (online).

1 $${E}_{\gamma }={\gamma }_{m}{A}_{m}+{\gamma }_{b}{A}_{b}$$

The magnetic energy in the ferrofluid droplet (*E*_*mag*_) is calculated as a function of the droplet volume (*Vol*_*ferro*_), magnetic permeability of vacuum (*μ*_*0*_), the external magnetic field evaluated at the centre of the droplet (*H*_*ferro*_), and the response of the droplet to the field (*M*_*ferro*_) as described in Eq. (2). This equation assumes that the ferrofluid droplet magnetization does not influence the external field and that the field within the droplet may be described by the value at the droplet center^61–63^. When an electromagnet is activated, the ferrofluid droplet becomes magnetized and produces a shift in this minimum energy as shown in Fig. 3a, altering the equilibrium distance between the droplets until droplets separate.

2 $${E}_{mag}=-\frac{1}{2}{\mu }_{0}{Vol}_{ferro}{M}_{ferro}{H}_{ext}$$

The response of the droplet with a varying magnetic field is predicted using the approach described within the Modeling Methodology section. The new minimum energy for the combination of *E*_*mag*_ and *E*_*g*_ is assumed to correspond to the distance between the droplet centres at equilibrium, reducing the total bilayer area between the droplets (*A*_*b*_, Fig. 3a).

Using data provided by the vendor Ferrotech (further detailed in Supplementary Table S2 online), the necessary gradient of the magnetic field predicted for droplet separation as a function of the droplet radius and energy of adhesion for both EMG509 and EMG507 can be reproduced (Fig. 3b). From these plots we can draw two conclusions. First, smaller droplets are more difficult to manipulate using magnetic fields given that the magnetic energy scales with volume and the interfacial energy scales with area. Second, for lower concentration ferrofluids (EMG509), complete saturation of the ferrofluid is reached prior to separation (shaded in black in Fig. 3b lower panel). Previous works have shown that solvents that produce more stable bilayers result in higher energy of adhesions^8,17^ and will require more force to separate. The use of the 1:1 hexadecane:silicone oil AR20 solvent in DIBs has been traditionally preferred since it reduces gravitational influences on the droplets and allows for the formation of stable networks^15^. However, these bilayers require additional magnetic force to separate given their strong adhesion energies (more details in the Modeling Methodology section). When compared to this mixture the 2:1 hexadecane:silicone oil AR20 combines both the advantages of reduced gravitational influences and overall network stability along with reduced forces necessary for droplet separation. Hence the use of EMG 507 in DIB networks constructed in a 2:1 hexadecane:silicone oil AR20 medium should allow for feasible droplet manipulation.

This was tested experimentally by examining the magnetic force that needs to be supplied to separate an EMG 507 droplet from a water droplet (with an interfacial membrane—Fig. 3c) with two compositions. Plotting the experimentally measured lipid bilayer area against the electromagnet current obtained from experiments for different oils and comparing the results against the predictions from the energetics model confirms the suggested mechanics for droplet separation (Fig. 3c,d). This further show how a higher energy of adhesion (resulting from an increased volume percent of silicone AR20 oil in the solvent phase) requires higher magnetic force (reflected through the current supplied to the electromagnets producing the field) for separation of the lipid bilayer. Examining the governing equations, several parameters of interest are provided. As mentioned previously, the field necessary to separate the droplets is governed by the energy of adhesion $\left(-\Delta F=2{\gamma }_{m} - {\gamma }_{b}\right)$ of the selected lipids and solvent (Table S1)^16,41^, which governs the depth of the energy well in Figs. 3a and 7b. The interfacial energy (*E*_*γ*_) scales with the surface areas of the droplets (*A*_*m*_, *A*_*b*_). The magnetic energy (*E*_*mag*_) scales with the ferrofluid properties (Supplementary Table S2 online and Fig. 7a) and ferrofluid volume (*Vol*_*ferro*_). Consequently, the ratio of the magnetic energy and interfacial energy will increase with the droplet radius *r*_*ferro*_. The droplet volume may be varied by adjusted by adjusting the duration and magnitude of the pressure pulse for deposition^27^, while the energy of adhesion is primarily a function of the selected continuous oil phase^16,17^. These outcomes indicate using EMG 507 ferrofluid in DIB-systems that are constructed in a 2:1 hexadecane:silicone oil AR20 solvent is the most feasible combination for droplet manipulation through magnetic forces. This combination is used for all following sections unless specified otherwise.

However, while the overall trends are similar, several discrepancies must be noted between the experimental cases and the model predictions. The model assumes the ferrofluid droplet remains spherical, while in reality the ferrofluid droplet elongates (Fig. 3c) in response to the magnetic field. Secondly, the increased density of the ferrofluid in comparison to the surrounding oil (Supplementary Tables S1–S2 online) causes the ferrofluid droplet to flatten. Third, the assumption that the magnetic field is uniform throughout the droplet may no longer be valid given the droplet dimensions. Fourth, the measurement of the monolayer tension of EMG 507 in oil was not feasible given that the ferrofluid both exhibits very low interfacial tensions with lipids and interacts with the dispensing syringe used for tension measurements. Values for the monolayer tension for ferrofluid droplets were consequently estimated to match the experimentally measured lipid bilayer areas without the magnetic field. The EMG 507 tension was estimated to be 0.88 mN m^−1^ in the 2:1 hexadecane: silicone oil AR20 and 0.84 mN m^−1^ in the 1:1 hexadecane: silicone oil AR20. These lower estimated monolayer tension values potentially align with modified monolayer formation expected with nanoparticle-lipid interactions at the oil–water interface^64^.

Still, this energetics model captures the mechanics associated with the magnetic manipulation of adhesive droplets and is provided for illustrative insights into how the magnetic reconfiguration may be tuned as the overall trends of the model and experimental results agree. With the behaviours predicted by the energetics model comparing favourably to experimental results, the model may be extended to observe scaling phenomena involved with ferrofluid droplet separation and guide experimental design. In the next section, we explore how these simple mechanisms may be combined in larger collections of adhered droplet clusters to approximate reconfiguration events observed in living tissues covered previously.

### Bioinspired shape-shifting within droplet networks

From the previous discussion and experimental results, we observe that ferrofluid droplets are able to push, pull, and rotate around their adhered non-magnetic partners. The adhesive connections between the droplets provide a form of elasticity, and networks of these droplets may behave as collections of linked particles capable of separating and reconnecting as they change their relative positioning within the collective tissue. Consequently, a single ferrofluid droplet may be used to exert forces on adhesive bridges of interconnected droplets, driving separation of existing bilayers and allowing the droplets to reform into new equilibriums.

The amount of magnetic input necessary for reconfiguration increases with the number of adhered droplets and their overall orientation relative to the direction of the magnetic field. After separation of the desired lipid membranes, the magnetic field is removed, and the droplets are allowed to settle into a new equilibrium. It should be noted that the process for new membrane formation is dependent on the two monolayers successfully expelling the solvent from between the lipids and beginning the wetting process^65^. This typically required between 0 and 2 min for bilayer formation in the experiments, but variation is still observed between cases.

Several two-dimensional collections of droplets are reproduced for reconfiguration events within networks using distributed ferrofluid droplets dispersed within the adhered network. Select droplets are anchored using hydrogel-coated glass pipettes, enabling separation of droplets by restricting the motion of the cluster. Each reconfiguration experiment is presented with a figure clearly showing both the original structure prior to magnetic perturbation and the produced structure after reconfiguration. In each of the cases, multiple samples were collected (n ≥ 3) where the current supplied to the electromagnets as well as the time it took the mechanism to reach completion was recorded. The results displayed in this work were rotated to show the same activation direction for the sake of consistency. Videos for several of these reconfiguration events are provided in the Supplementary Information online at 4 × speed.

**Figure 4:**
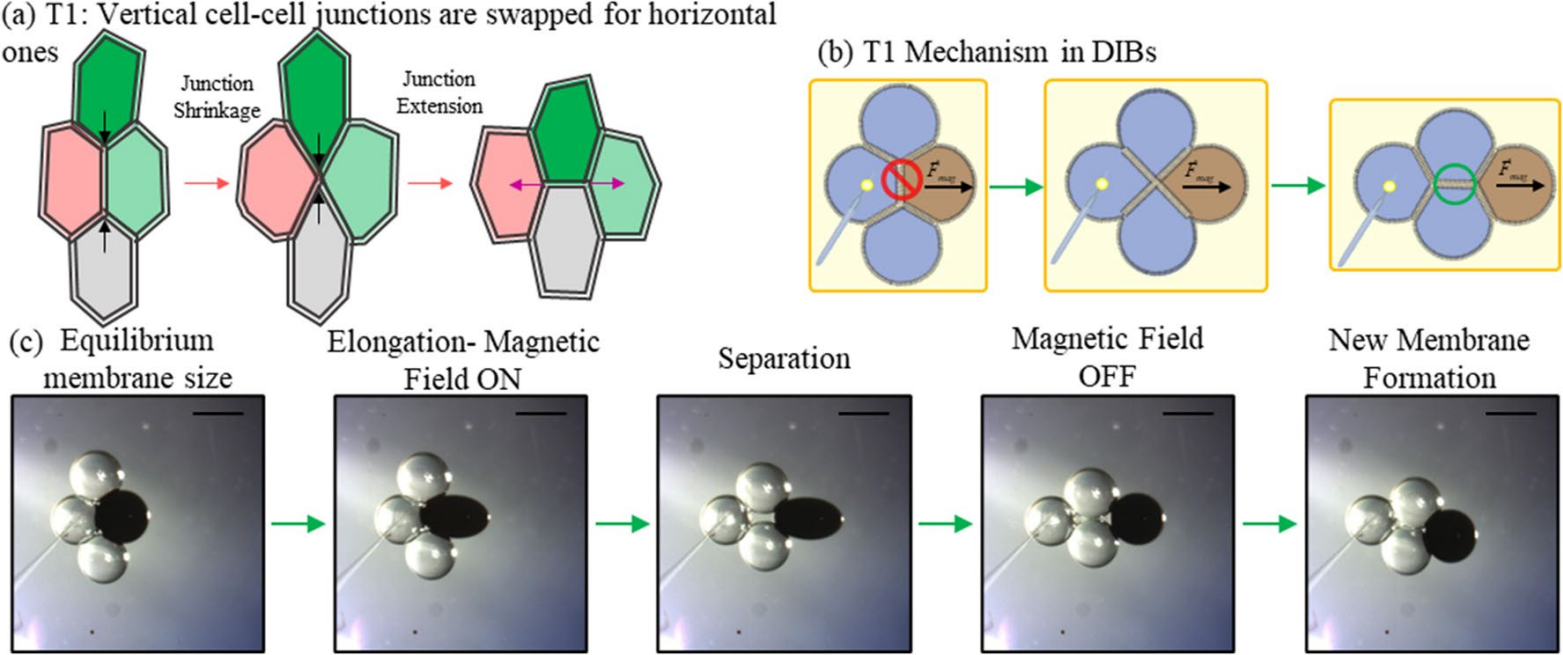
Reconfiguration events in DIB networks. (**a**) The T1 event is a reconfiguration mechanism observed in cellular tissues. Colors are intended to aid in tracking relative cell location. (**b**) Reconfiguration in DIBs mediated through membrane separation/reformation may be produced using magnetic fields. (**c**) Experimental images (top view) obtained when magnetically activating a T1 event in a set of four adhered microdroplets. Scale bar represents 600 μm. The ferrofluid droplet (EMG 507) is pulled towards the activated electromagnet. Once the vertical junction membrane is separated, the magnetic field is turned OFF. After being given sufficient time (around 1 min) a new horizontal membrane junction is formed, similar to the T1 mechanism. Schematic representations in (**a**) and (**b**) were created by the authors using Microsoft PowerPoint.

The distribution of bilayers in a simple four-droplet cluster may be rearranged through magnetically-induced T1 events (Fig. 4a). In this configuration (Fig. 4b,c), a single ferrofluid droplet separates an existing vertical lipid bilayer and replaces this with a horizontal one. One aqueous droplet is fixed into place with a hydrogel anchor and the ferrofluid droplet is pulled away from this anchor using a magnetic field, separating the bilayer between the ferrofluid and the anchor. As the ferrofluid droplet detaches, the neighbouring droplets are pulled together after rotating along their neighbouring surfaces and form a new horizontal lipid membrane together. Such event required a supplied current for electromagnets ranging from 1.5 to 3.5 A for an estimate duration of 4–13 s (followed by an approximate duration of 60 s for the formation of new interfacial lipid membranes).

**Figure 5:**
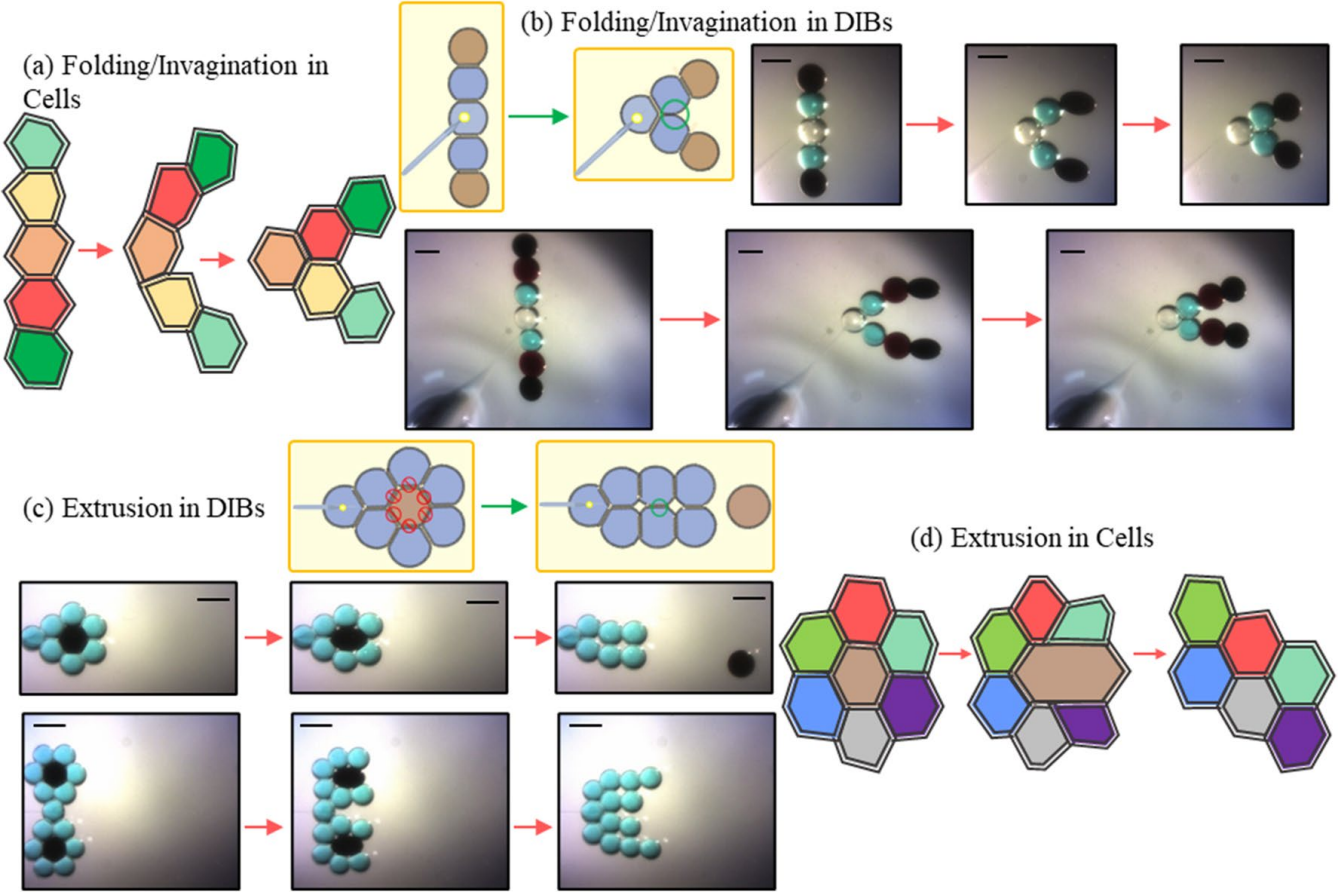
Summary of additional reproduced reconfiguration mechanisms in DIBs. The folding/invagination (**a**) action uses ferrofluid droplets (**b**) to pinch droplets together about a hydrogel anchor. A first case uses two EMG 507 droplets while a second case shows a folding mechanism enabled by using two types of ferrofluids with different concentrations of magnetic particles. The extrusion mechanism (**c**) extracts the ferrofluid droplet from the center of a hexagonal packed structure to form new interfaces. This has been accomplished in (**d**) where a single a double extraction is performed as shown by the experimental images' series (using EMG 507). DIB structures were formed with aqueous droplets (blue and white colors), EMG 507 (black color) and EMG 509 (brown color) droplets. Scale bars represent 600 µm each. Schematic representations were created by the authors using Microsoft PowerPoint.

Folding (Fig. 5a) is accomplished by attaching two ferrofluid droplets to the end of a droplet chain and anchoring the chain at the desired folding point. As shown in Fig. 5b, the ferrofluid droplets begin moving towards the activated electromagnet. The adhesive forces along the chain cause them to begin rotating the chain inwards towards the opposite side. Eventually the droplets rotate into contact and form a new lipid membrane. This is the simplest reconfiguration mechanism as it does not require the separation of existing membranes and may be accomplished with minimal magnetic forces. The total movement required a magnetic energy per ferrofluid droplet ranging from 2.5 to 3.5 A supplied current per electromagnet and took an average of 16 s for separation with an additional average of 21 s for the new interfaces to form after the droplets were pulled into contact.

Extrusion (Fig. 5d) is replicated by placing a ferrofluid droplet (EMG 507) at the centre of packed hexagonal DIB structure as shown in Fig. 5c. When a magnetic field is applied, the central ferrofluid droplet exerts local forces on the rest of the structure. As the magnetic droplet moves and deforms at the centre, the bilayers bordering it are gradually separated. As the droplet moves out of the centre of the cluster, attached droplets are repositioned and pulled into contact. Once the last bilayer is separated, the magnetic droplet is expelled from the DIB structure into the surrounding oil environment. Post- extrusion, the remaining non-magnetic droplets are pulled into contact with each other and form new lipid membranes. The total movement required a 3.5–5.0 A supplied current for the electromagnet and needed an average of 17 s for separation with an additional minute for the new interfaces to completely form.

Each of the described reconfiguration mechanisms may be combined in larger networks and executed simultaneously, such as a combined extrusion and folding event (Supplementary Video online). Experimental demonstrations are provided in Fig. 5b,c. These may be accomplished using varying ferrofluid compositions (Supplementary Table S1 online), pre-set structures, and the same anchor scheme for providing fixed boundary conditions as before. Each of these figures presents the original structure, distorted structure during perturbation, and the new equilibrium structure produced after disabling the external magnetic field.

### Application of magnetically enabled reconfiguration of bilayers structure

As noted earlier, the rearrangement of the droplets within the DIBs also changes the nature of the lipid membranes, as the properties of the lipid membranes at the intersection of the droplets are determined by the droplet pair. Consequently, it is feasible to reconfigure the droplets to dramatically change their functionality as demonstrated here.

**Figure 6:**
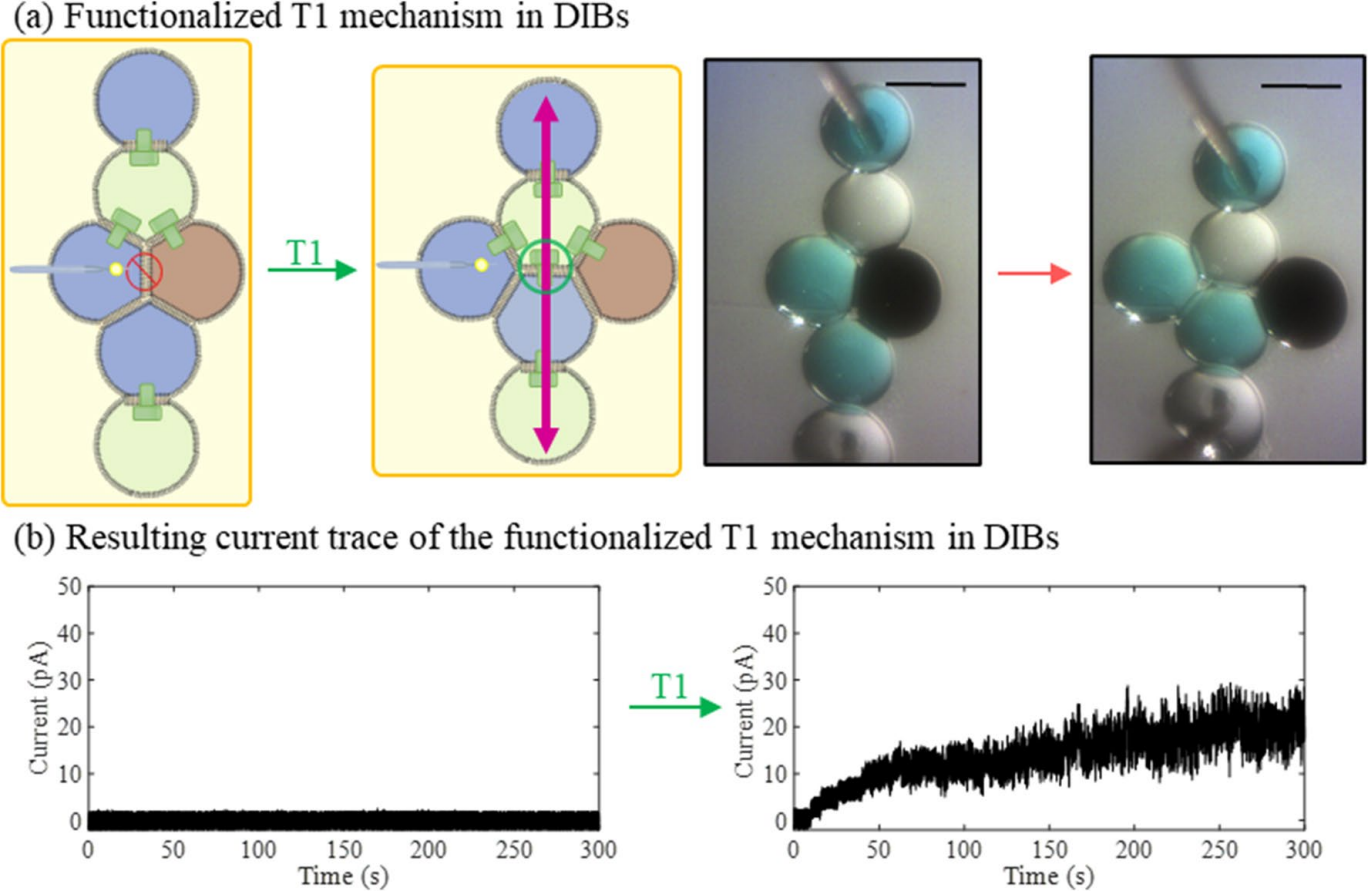
(**a**) Schematic representation and experimental results of the functionalized T1 neighbor exchange mechanism DIB network (formed with aqueous droplets (blue color), αHl infused aqueous buffer (2 µg/ml—white color) and EMG 507 (black color) droplets). Scale bars represent 600 µm each. (**b**) Current traces obtained for a constant + 100 mV DC applied across the DIB networks shown respectively in (**a**) for the variation in αHL insertion activity upon the magnetic induction of a T1 rearrangement mechanism. Changes in the current reflect changes in the overall conductance of the tissue through insertion of the PFTs into individual bilayers. All measurements were recorded in voltage clamp mode at a sampling frequency of 10 kHz and filtered at 1 kHz (using the embedded low-pass Bessel filter − 80 dB/decade). Post-acquisition, data was filtered at 500 Hz using a fourth-order Butterworth low-pass filter in MATLAB. Scale bars represent 600 μm each. The schematic representation in (**a**) was created by the authors using Microsoft PowerPoint.

One of the simplest methods for enabling droplet–droplet exchange involves pore-forming agents such as the pore forming toxin (PFT) alpha-hemolysin (*α*Hl). PFTs insert into lipid membranes without conformational states and create large non-gating channels^66^, enhancing the diffusive exchange between the compartments^67^. As a proof of the capabilities of magnetically infused DIB structures in generating functional reversible structure rearrangement processes, we focused on enabling transport through selectively infusing *α*Hl into droplet reconfiguration in a way that generates conductivity post magnetically triggered intercalation event. Figure 6 shows the effect of a magnetically guided T1 intercalation event on the measured conductivity of a DIB network. After the droplets containing αHl are aligned between the electrodes, pores are formed within the newly formed membranes. This permits the passage of ions across all membranes between the two electrodes as showcased by the increase in measured current with 100 mV applied between the electrodes (Fig. 6b). Since the exchange across the bilayers and the properties of the bilayers themselves formed using this approach are largely dictated by the droplet compositions^32,68–70^, this new method for driving reconfiguration of the droplets may be used to establish new capabilities for the synthetic tissues.

## Conclusion

This research produced droplet-based materials capable of swapping between functional configurations using magnetic forces. This was made possible by reconfiguring the underlying droplet arrangements through separation and reformation of the interfacial membranes, taking advantage of the unique composition mechanism of DIB membranes. First, the mechanics of droplet separation through magnetic forces was investigated using an energetics model and experimental data to estimate the magnetic field necessary for droplet separation as a function of droplet dimensions, ferrofluid properties, and selected solvents and lipids. The model predictions were compared against experimental results for validation and used to select an optimal combination of ferrofluid and solvent properties. Next, the concept was then extended to the manipulation of ferrofluid droplets embedded within DIB clusters. The ferrofluid droplets are capable of exerting forces on their adhered neighbours, and can separate then recombine surrounding droplets, reshaping the membranous architecture. Several reconfiguration events based on cellular intercalation events were demonstrated, and the findings were applied to larger networks as well. Finally, the technique was combined with selectively distributed pore forming toxins to produce a conductive pathway between two electrodes using a T1 rearrangement event.

The presented research emphasizes reconfiguration of the droplets comprising the material rather than overall shape changes, or shape-shifting. Altering the relative droplet locations within DIB tissues produces changes in the internal membranous architecture, and it is possible to adjust the internal droplet–droplet exchange as a result. The results demonstrate a new approach for designing these bioinspired droplet networks with multiple functionalities that may be enabled and disabled as needed through adaptation of the droplet arrangements.

## Methodology

Additional detailed experimental protocols and methodologies are provided in the Supplementary Information online for replication. Brief overviews of the techniques are provided here for convenience.

### Materials

Aqueous solutions were prepared with a standard buffer solution with (250 mM Potassium Chloride (KCl)) and (10 mM 3-(N-morpholino) propanesulfonic acid (MOPS)) added yielding a pH of 7.0. This solution was then used to produce solutions with and without dye for visualization, with and without lipids using standard techniques^17,41^, and with or without the pore-forming toxin alpha hemolysin for enabling diffusion between neighbouring droplets. Aqueous ferrofluid solutions were acquired from Ferrotec, including EMG 507 and EMG 509. KCl and MOPS were added to match the osmolality of the buffer solution and produce a neutral pH while watching for settling and aggregation of the nanoparticles. EMG 507 offers a stronger magnetic response in comparison to EMG 509, with the penalty of a substantially higher density and nanoparticle concentration. As a general rule the magnetic properties of the ferrofluid scale with the nanoparticle concentration by volume, providing a mechanism for enhancing the magnetic responsivity of the droplets. The selected oil phase consisted of various mixtures of hexadecane: silicone oil AR20 intended to produce a favourable balance of interfacial tensions^8,17,71^. Mixtures of 1:0, 1:1, and 2:1 were studied and the 2:1 mixture was found to both increase the bilayers’ stability while facilitating the magnetic manipulation of droplets, and separation/reformation of lipid membranes^8,16^, as determined through a combination of pendant drop tensiometry and visual measurements of bilayer tension. Lipids were added where specified. Aqueous lipid solutions underwent standard extrusion and sonication techniques prior to use.

### Methods

An electromagnet-based manipulation system was assembled to remotely control magnetically susceptible droplets. A schematic representation of this setup is presented in Fig. 2. Droplets of varying solutions are deposited on the working stage using a pneumatically-driven 3D-droplet printer described previously, modified to support multiple solutions^27^. After the droplets are deposited, they are manipulated using magnetic forces to produce the desired reconfiguration events. Four solenoids are mounted in the cardinal directions and selectively enabled through a LABVIEW interface. Designated anchor droplets are fixed in place using glass rods coated in a hydrogel, providing fixed droplets for boundary conditions and enabling reconfiguration rather than translation of the network through magnetic forces. Electrical measurements are conducted through standard electrophysiology practices using two silver/silver-chloride wires connected to a patch-clamp apparatus. All measurements are conducted in a vibration isolation table within a Faraday cage, and images are recorded using a CCD camera attached to a zoom microscope.

### Modeling methodology

#### Overview of the model

To approximate the response of DIBs to an externally supplied magnetic field, the summed contributions from interfacial energy *E*_*γ*_ and magnetic energy *E*_*mag*_ are plotted, and the new equilibrium is found. The force on the droplets *F* may be approximated as the gradient of the two values as described in Eq. (3).

3 $$\overrightarrow{F}\approx -\overrightarrow{\nabla }({E}_{mag}+{E}_{\gamma })$$

In this model we assume a 1-D axisymmetric behavior with droplet motion only considered in the *z* direction. The condition for equilibrium where *F*_*z*_ = 0 may be written as a balance of the magnetic contributions and interfacial contributions in Eq. (4):

4 $$\frac{\partial {E}_{mag}}{\partial z}=-\frac{\partial {E}_{\gamma }}{\partial z}$$

This corresponds to any local minimums in the energy with respect to *z*. The interfacial energy *E*_*γ*_ is a function of the monolayer and bilayer tensions (*γ*_*m*_, *γ*_*b*_) multiplied by their respective areas (*A*_*m*_, *A*_*b*_).

5 $${E}_{\gamma }={\gamma }_{m}{A}_{m}+{\gamma }_{b}{A}_{b}$$

The summed interfacial energy for the DIB is calculated by assuming that the adhered droplets may be approximated as spherical caps. This is accomplished by defining the droplets as two entities with fixed volumes and a prescribed distance between their centers as shown in Fig. S1. If the droplets radii *R* overlap, it is assumed that a bilayer is formed at their interface whose radius *a*_*m*_ may be calculated as a function of the droplet radius *R* and the height of the spherical cap *h*. However, the volume of the spherical cap must be added back into the original droplet volume and the apparent radius *R* must be recalculated which further influences *a*_*m*_. The correct solution enforcing a fixed droplet volume is found through iteration.

Once values for *R*, *h*, and *a*_*m*_ are satisfactorily determined for the prescribed distance between the centers, the areas for the monolayer *A*_*m*_ and bilayer *A*_*b*_ may be calculated using Eq. (6).

6 $$A_{b} = \pi a_{m}^{2} \;\;\;\;\;\;A_{m} = 4\pi R^{2} - \pi \left( {a_{m}^{2} + h^{2} } \right)$$

**Figure 7:**
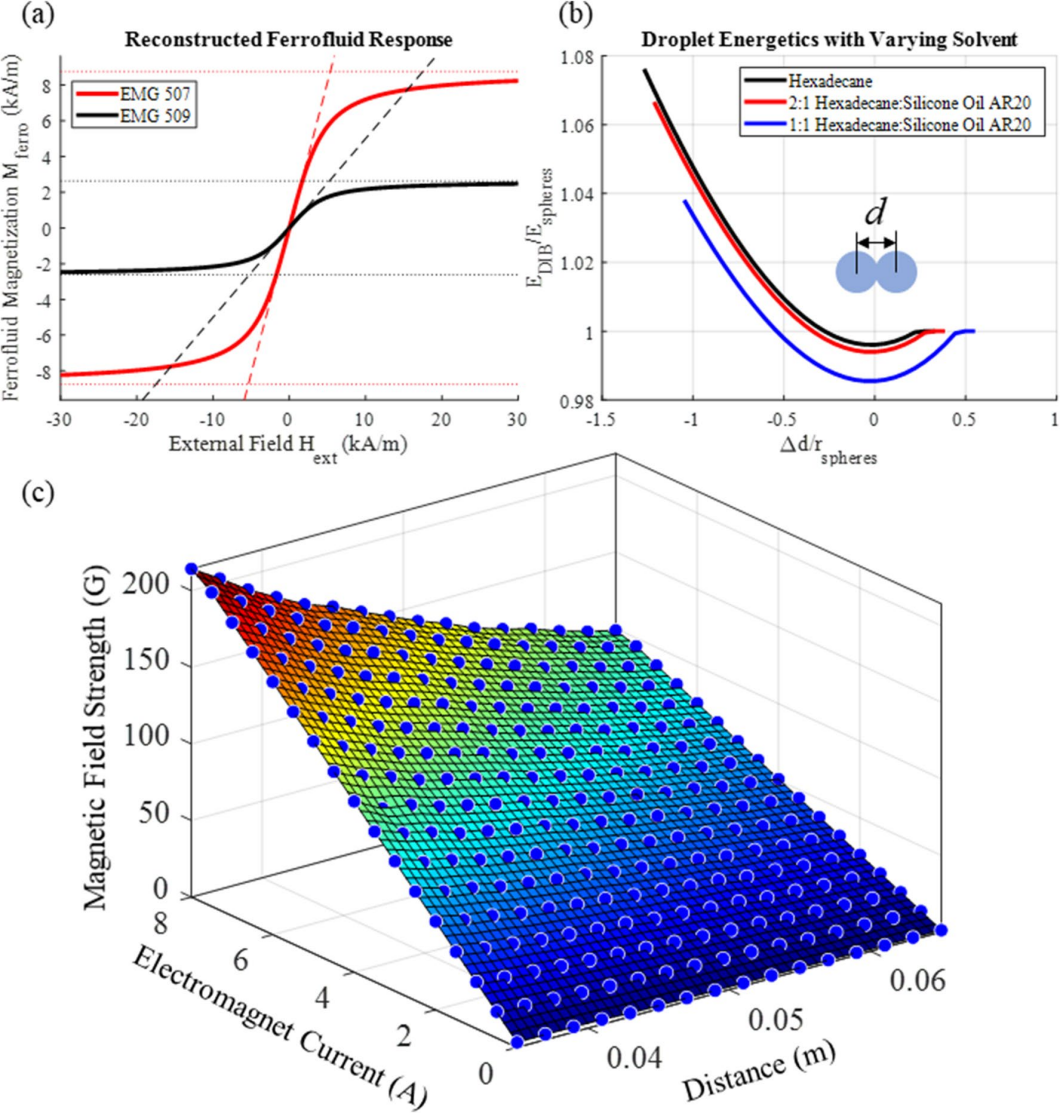
Model setup. (**a**) Plots of the ferrofluid magnetization *M*_*ferro*_ (EMG 507 and EMG 509 water-based series) with an external field *H*_*ext*_ for the two selected ferrofluids including saturation effects. The dashed lines are the linear magnetization response provided by *χ*_*ferro*_, and the dotted lines are the saturation limits. (**b**) Plots of the interfacial energy (*E*_*γ*_) for a pair of adhered droplets as a function of the normalized distance between their centers offset by the equilibrium distance. (**c**) Quantified electromagnet response to supplied current.

Multiplying these values by their prescribed interfacial tensions produces the total droplet interfacial energy (Eq. (5)). Plotting this energy as a function of the distance between the droplet centers for varying solvents produces Fig. 7b. This is similar to results predicted by a Surface Evolver model in a previous work^8^.

Notably, the minimum energy matches the equilibrium contact angle prescribed by Young’s law (Eq. (7)). This provides a description of the droplet–droplet interactions and the elasticity afforded by the adhesive emulsion that resists separation. The stronger the energy of adhesion, the greater the necessary magnetic field is necessary to separate the droplets.

7 $$2{\gamma }_{m}cos{\theta }_{m}={\gamma }_{b}$$

Values for the interfacial tensions used in are provided in Supplementary Table S1 online. These values were obtained using pendant drop tensiometry for the monolayer tension *γ*_*m*_ combined with measured angle of contact through microscopy for the bilayer tension *γ*_*b*_ and Eq. (7). For each case the aqueous phase was 250 mM KCl with 10 mM MOPS. Each measurement was repeated five times and standard deviations are provided.

The magnetic energy in the ferrofluid droplet *E*_*mag*_ is a function of the droplet volume *Vol*_*ferro*_, magnetic permeability of vacuum *μ*_*0*_, the external magnetic field evaluated at the center of the droplet *H*_*ferro*_, and the response of the droplet to the field *M*_*ferro*_ as described in Eq. (8).

8 $${E}_{mag}=-\frac{1}{2}{\mu }_{0}{Vol}_{ferro}{M}_{ferro}{H}_{ext}$$

The external field *H*_*ext*_ is assumed to remain constant across the droplet interior. The droplet response is calculated as a function of this external field. The saturation *M*_*sa*_ and initial susceptibility *χ*_*ferro*_ variables are provided in Supplementary Table S2 online. The response of the ferrofluid may be approximated through a Langevin function *L*(*α*)^61,72^,accounting for saturation effects when all nanoparticles are aligned with the external magnetic field. As a result, the response *M*_*ferro*_ will be capped by the saturation magnetization *M*_*sat*_ of the ferrofluid shown in Eq. (9).

9 $${M}_{ferro}={M}_{sat}L\left(\alpha \right)={M}_{sat}\left(\mathit{coth}\left(\alpha \right)-\frac{1}{\alpha }\right)$$

The Langevin function may be approximated through a Taylor series as described in Eq. (10) ^72^:

10 $$L\left(\alpha \right)\approx \frac{\alpha }{3}-\frac{{x}^{3}}{45}+\dots$$

We use the linear approximation for *L*(*α*) to determine the coefficient *α* using the initial linear relationship between the ferrofluid magnetization *M*_*ferro*_ and external field *H*_*ext*_ as defined by the initial susceptibility *χ*_*ferro*_ as described in Eq. (11).

11 $$M_{ferro,linear} = \chi_{ferro} H_{ext} = M_{sat} \left( {\frac{\alpha }{3}} \right)\;\;\;\;\;\;\alpha \approx 3\frac{{\chi_{ferro} H_{ext} }}{{M_{sat} }}$$

Using the ferrofluid properties provided by the vendor in Supplementary Table S2 online it is possible to reconstruct the ferrofluid response to an external field for the two ferrofluids as shown in Fig. 7a. The predicted magnetization curves clearly fit with the provided saturation values (maximum bounds) and linear regions (fit of the slope about *H*_*ext*_ = 0).

The magnetic field produced within the electromagnet may be predicted by Eq. (12) as a function of the maximum permeability of the soft iron core *μ*_*r,core*_, supplied current *i*_*mag*_, turns per length *N*/*L*, and permeability^72^. Consequently, the magnetic field will scale approximately with the supplied current.

12 $${B}_{\infty }\approx {\mu }_{r,core}{\mu }_{0}\frac{N}{L}{i}_{mag}$$

The electromagnet configuration for examining droplet separation may be seen in Fig. 3. The droplets were centered with the electromagnet (x = 0, y = 0) at a set distance from the surface of the electromagnet. The electromagnet dimensions and properties are provided in Supplementary Table S3 online.

The magnetic field *B* was measured experimentally using a DC Gaussmeter (GM1-ST, AlphaLab). This measurement was taken in 0.5 A increments from 0 to 8 A, the available range from the power supply. The probe location was varied in 2 mm increments using a manual micromanipulator (Siskiyou, Grants Pass, OR), ranging from 3.5 cm to 6.5 cm from the magnet face, and the measurements were repeated 3 times. Values were recorded at the center of the produced magnetic field (x,y = 0, Fig. S2). These values were then fit to a 3^rd^ order polynomial surface (poly33) using MATLAB to interpolate the magnetic field for the droplet distance from the electromagnet and input currents as seen in Fig. 7c.

These equations do not account for deviations away from spherical droplet shapes, influence of droplet magnetization on the external field, or phenomena such as friction and solvent viscosity which will further influence the droplet dynamics^73^. However, they do capture the general mechanics responsible for magnetic manipulation of DIBs and may be used to guide experimental design. The response of the droplet with a varying magnetic field is predicted using the approach described previously. The new minimum energy for the combination of *E*_*mag*_ and *E*_*γ*_ is assumed to correspond to the distance between the droplet centres at equilibrium, reducing the total bilayer area between the droplets (*A*_*b*_, Fig. 3c).

## Supplementary Information

Supplementary Information 1.

Supplementary Legends.

Supplementary Video S1.

Supplementary Video S2.

Supplementary Video S3.

Supplementary Video S4.
