# Supplementary material for: Enhancing membrane-based soft materials with magnetic reconfiguration events
Source: Sci Rep. 2022 Feb 1;12:1703. doi: 10.1038/s41598-022-05501-7 (PMC8807651; doi:10.1038/s41598-022-05501-7)
Supplement: Supplementary file 1 — Supplementary Information 1. [file 41598_2022_5501_MOESM1_ESM.pdf]

Supporting Information

Enhancing Membrane-Based Soft Materials with Magnetic Reconfiguration Events

*Makhoul-Mansour, Michelle M.; El-Beyrouthy, Joyce B.; Mao, Leidong; Freeman, Eric C.**

Supplementary information includes a detailed overview of the experimental methodology, materials used and additional details.

Experimental Methodology

Materials

Background Aqueous Buffer Solution

The main aqueous buffer solution was prepared by adding ((250 mM) Potassium Chloride (KCl, Sigma-Aldrich, St. Louis, MO)) and ((10 mM) 3-(N-morpholino) propanesulfonic acid (MOPS, Sigma-Aldrich, St. Louis, MO)) to deionized water yielding roughly a pH of 7.0 as measured and was subsequently used for liposomes hydration. A commercial food dye (Kroger assorted water-based food colouring kit, purchased from a local grocery store) was used at a weak concentration ($\leq 1\mu$l dye.ml^{-1} of solution) to induce a blue colour in droplets for illustrative purposes only.

Aqueous Lipid Solutions

Zwitterionic lipids (1,2-diphytanoyl-sn-glycero-3-phosphocholine (DPhPC, Avanti Polar Lipids, Alabaster, AL) were first dispersed at the desired concentration in chloroform (Sigma-Aldrich, St. Louis, MO) yielding clear solutions. The organic solvent was then evaporated through exposure to a dry argon stream under a fume hood for several minutes. The resulting lipid film was then further dried by placing the vial in a room-temperature vacuum chamber for a minimum of 6 hours. Dried lipid films were consequently rehydrated by adding the previously described aqueous buffer solution ((250 mM) KCl and (10 mM) MOPS) yielding a final lipid concentration of (2.5 mg.ml^{-1}). Afterwards, the lipid-mixtures were homogenized by gently stirring them using a vortex agitator. These solutions subsequently underwent several thaw-freeze cycles 6 cycles and were afterwards stored at 2 °C. Upon usage, the lipid solutions were sonicated using a probe tip sonicator (2 mm standard probe tip with a maximum oscillation amplitude of 200 μm, from QSONICA Q55 Probe Tip Ultrasonicator, QSONICA, Newtown, CT) until a clear consistency was achieved and no lipids aggregations were observed (30 W cycles of 2 minutes for a minimum of 5 cycles). The tip of a sonicator was roughly positioned in the middle of the lipid dispersion ensuring a uniform energy dissipation within the vials. This preparation process delivers a high-energy input into lipid suspensions and ensures that the resulting vesicles are optimal for DIB membrane formation.

Alpha-Hemolysin Infused Solutions

A concentration of (2 μg.mL^{-1}) of wild-type alpha hemolysin αHl from Staphylococcus aureus (Sigma-Aldrich, St. Louis, MO) was added to the previously described buffer solution and stored at 2°C. This oligomerized PFT heptamers introduce themselves spontaneously into lipid bilayers to form mushroom-shaped pores that allow small molecules to pass through the membrane[1]. Each αHl insertion event is usually marked by a stepwise increase in the measured transmembrane current (for a single lipid membrane, when multiple lipid membranes are monitored insertion events are seen as an increase in the measured current- not necessarily stepwise) [2,3].

Aqueous Ferrofluid Solutions

EMG 507 and EMG 509 (EMG series water-based ferrofluid, Ferrotec, Santa Clara, CA), commercial water based ferrofluids with a magnetic particle concentration of 2% and 0.6% respectively (by volume) were used throughout this work. Parameters for each ferrofluid are

provided in **Table S2**. 250 mM KCl and 10 mM MOPS salts were added to both ferrofluid solutions. EMG 507 has a distinctive black color reflecting its higher concentration of magnetic nanoparticles while EMG 509 has lighter consistency (lower density) and a brown pigment reflecting its lower magnetic nanoparticles concentration. All ferrofluid solutions were systematically and periodically sonicated to ensure homogeneity using a probe tip sonicator (the same 2 mm standard probe tip with a maximum oscillation amplitude of 200 µm, from QSONICA Q55 Probe Tip Ultrasonicator, QSONICA, Newtown, CT). Sonication was held in 30 W cycles of 2 minutes each; on average, 4 cycles were required resulting in a more homogenous suspension of magnetic nanoparticles in the injected microdroplets. A uniform distribution of magnetic nanoparticles yields by extension a more uniform magnetic field force distribution upon the application of magnetic fields.

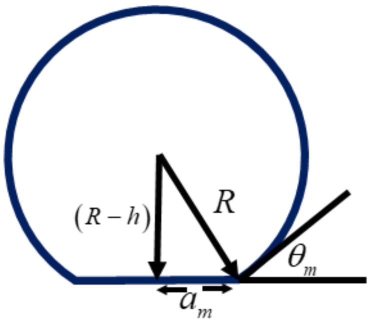

Supplementary Figure 1 – Spherical cap approximation for the droplet geometry and produced membrane radius a_m, apparent droplet radius R, spherical cap height h, and angle of contact q_m.

Lipid-Oil Solution

When the lipid-out technique was used, Zwitterionic lipids (1,2-diphytanoyl-sn-glycero-3-phosphocholine (DPhPC), Avanti Polar Lipids, Alabaster, AL) were typically suspended in a 2:1 (volume ratio) mixture of hexadecane and silicone oil AR20 (both Sigma-Aldrich, St. Louis, MO) at a concentration of (0.5 $mg.ml^{-1}$). These solutions were bath-sonicated for 15 minutes (Elmasonic S100h Ultrasonic sonicator, Elma Schmid Bauer GmbH, Gottlieb-Daimler-Straße Singen, Germany) and afterwards stored at 2 °C. Prior to each use, the solutions were refreshed by sonicating them again for 15 minutes. This mixture was found to both increase the bilayers' stability while facilitating the magnetic manipulation of droplets, and separation/reformation of membranes[4,5].

Supplementary Table 1 – Interfacial Tensions for Varying Solvents

**Mixture**	**Monolayer Tension (γ_m)**	**Bilayer Tension (γ_b)**	**Energy of Adhesion ($-\Delta F$)**	**Density**
Hexadecane (DPhPC, Lipid-In, 2.5 mg/mL)	1.05 (±0.05) mN/m	1.83 (±0.11) mN/m	0.28 mN/m	774 kg/m^3
2:1 Hexadecane: Silicone Oil AR20 (DPhPC, Lipid-Out, 0.5 mg/mL)	1.10 (±0.03) mN/m	1.85 (±0.08) mN/m	0.36 mN/m	853 kg/m^3
1:1 Hexadecane: Silicone Oil AR20 (DPhPC, Lipid-Out, 0.5 mg/mL)	1.06 (±0.04) mN/m	1.59 (±0.07) mN/m	0.53 mN/m	892 kg/m^3

Methods

Experimental Setup (Solenoid-Based Magnetic Manipulation and Pneumatic Droplet Injections Systems)

An electromagnet-based manipulation system was designed to remotely control ferrofluid droplets. Four solenoids (originally purchased as air core solenoids, Ward's Science, VWR, Radnor, PA) with EFI Alloy 50 cores (2.5x7.5 inches cylindrical cores, Ed Fagan, Franklin Lakes, NJ) are mounted in parallel pairs in the x and y directions offering a precise control

over the planar position of the magnetic droplets (the distance between each solenoid pair is 12 cm while the distance from each solenoid to the center of the dish is 6 cm). These solenoids are powered through the 9111 BK Precision 60 V Multirange DC Power Supply (B&K PRECISION North America, Yorba Linda, CA). The magnitude of the magnetic energy provided by electromagnets can be controlled through the intensity of the current or voltage supplied to them. In order to move a droplet in 2D, using multiple solenoids at a time and precisely controlling the sequence in which a solenoid is magnetized are crucial. For this sake, power received by the electromagnets was interactively regulated through an ARDUINO MEGA 2560 microcontroller connected to an 8-channel relay module for ARDUINO, driven by a LABVIEW user interface. Such control scheme enabled us to actively control the direction of the applied magnetic field at a desired rate.

Larger DIB structures are constructed using the pneumatically-driven 3D-droplet printer[6]. In short, a capillary tube (glass tubes, 1.1mm x 10 cm, World Precision Instruments Inc., Sarasota, FL pulled using the World Precision Instruments, P-1000, Sutter, Sarasota, FL) filled with the desired aqueous solution is connected to the printing needle and pressure clamp (HSPC-2-SB, ALA Scientific Instruments, Farmingdale, NY) using silicone tubes. Another ARDUINO computer-controlled microcontroller is used to send voltage pulses to the HSPC which translate into an applied pressure to the operating liquid into the tube. The position of the capillary tube (and consequently that of the printed aqueous droplets) is controlled using a three-axis motorized micro-step manipulator (MCL3). The size of the droplets is dictated by the amplitude of the applied pressure, the duration of pressure application as well as the size of the glass tube opening. Adequate combination settings producing droplets with preset dimensions were calibrated for at the beginning of each experimental trial.

Pulled glass tubes (4 inches glass rods, 1.0mm x 10 cm, World Precision Instruments Inc., Sarasota, FL) were introduced into the dish to act as anchors for bilayer separation and reformation. The tips of these anchors were coated with agarose (3% by mass EZ Pack Agarose LE, Molecular Biology Grade, Benchmark Scientific, Sayresville, NJ) used to hold certain critical droplets in place. When electrical recordings are performed, droplets (~200-300 μm radius) were systematically injected on agarose (3% by mass EZ Pack Agarose LE, Molecular Biology Grade, Benchmark Scientific, Sayresville, NJ) coated silver/silver chloride (Ag/AgCl) electrodes (125 μm in diameter, Good Fellow, Coraopolis, PA). Both electrodes as well as anchors' positions were controlled through a three-axis manual micromanipulator (Siskiyou, Grants Pass, OR). Images/videos of DIB networks were acquired using a CCD camera (high sensitivity DCC1645C-HQ, Thorlabs, Newton, NJ) mounted on an inverted microscope. Cross-sectional images were also acquired using a CCD camera (high sensitivity DCC1240C, Thorlabs, Newton, NJ) to which zoom lenses (6.5X zoom lenses with a 0.7−4.5× magnification range, Thorlabs, Newton, NJ) were attached. Note that in all experiments, oil dishes were treated with sigmacote siliconizing reagent for glass and other surfaces (Sigma-Aldrich, St. Louis, MO) to prevent droplets from sticking to the surface.

Supplementary Table 2– Provided Ferrofluid Magnetic Properties (as provided by FerroTec)

**Parameter**	**EMG 507**	**EMG 509**
Initial Magnetic Susceptibility (c_{ferro})	1.63	0.5
Saturation Magnetization	110 Gauss	33 Gauss
Density	1120 kg/m^3	1030 kg/m^3
Magnetic Particle Concentration	2% vol	0.6% vol

<u>Application of a Variable Magnetic Field on a Single DIB-Experimental Approach</u>

Using the magnetic manipulation stage described previously, a water microdroplet is deposited in the lipid-oil mixture and held in place using a fine-pulled glass tube (as described

in the previous section). A lipid bilayer is formed by manually pushing an EMG 507 ferrofluid droplet into contact with the aqueous buffer droplet (using a second finely pulled glass rod). A magnetic field, perpendicular to the lipid membrane is then applied; top and side view images of the bilayer are acquired (once the DIB reaches an equilibrium size post magnetic field application). The intensity of the magnetic field is gradually amplified by augmenting the current supplied to the solenoid (in 0.5 A steps ranging from 0 A to 7.0 A) and the bilayer's response recorded once it reaches an equilibrium size. 3 samples were recorded and the data analyzed and averaged.

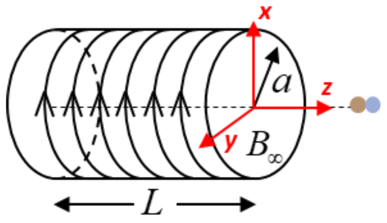

Supplementary Figure 2 – Definitions for the electromagnet configuration for manipulating a single DIB.

Electrophysiological Measurements

The obtained current traces were recorded in a Faraday cage (Thorlabs, Newton NJ) in voltage clamp mode (Whole Cell β=1) using the AXOpatch 200B patch clamp amplifier and the Digidata 1550 data acquisition system (Molecular Devices, Sunnyvale, CA) at a sampling frequency of 10 kHz and then filtered at 1 kHz using the embedded low-pass Bessel filter (−80 dB.decade^{-1}). Post-acquisition, a 500 Hz fourth-order Butterworth low-pass filter was applied in MATLAB for visualization purposes. This approach was used previously[7-9] and was employed as well for all electrical recordings here unless specifically stated otherwise.

Supplementary Table 3 – Experimental Parameters for Magnetic Separation of the Droplets

Variable	Value	Source
Solenoid Length (L)	18.4 cm	Measured
Solenoid Radius (a)	6.35 cm	Measured
Solenoid Turns (N)	~100	Measured
Ferrofluid Distance from Electromagnet	~5 cm	Variable
Monolayer Tension (γ_m)	1.10 mN/m	Measured
Bilayer Tension (γ_b)	1.85 mN/m	Measured
Droplet Radius (r_{ferro})	650 mm	Approximate
Electromagnet Maximum Relative Permeability ($\mu_{r,core}$)	100,000	Manufacturer (EFI Alloy 50, Ed Fagan)
Electromagnet Saturation Inductance	14,500 Gauss	Manufacturer (EFI Alloy 50, Ed Fagan)
Electromagnet Coercive Force	4.77 A/m	Manufacturer (EFI Alloy 50, Ed Fagan)

Pendant Drop Tension Measurements and Contact Angle Measurements

Monolayer tension measurements were obtained using the pendant drop technique with the open-source software OpenDrop[10]. The experimental apparatus consists of a calibrated needle containing the solution suspended within an oil reservoir inside a 3 mL glass cuvette. A droplet is suspended from the needle within the oil, and as the lipid monolayer forms at the oil-water interface the droplet will gradually adopt a pendant shape. Recordings are taken using a CCD camera attached to a zoom lens. Frames are extracted using MATLAB and used to estimate the monolayer tension as a function of time. The equilibrium monolayer tension γ_m is obtained and provided for varying solvent in **Table S1**.

The bilayer tension is obtained using the angle of contact between two adhered droplets. DIBs are formed for each solvent and images at exported again to MATLAB. The findcircles() algorithm is used to detect the outer edges of each droplet and their respective centers. These coordinates are used then to find the intersection points for the membrane and calculate the angle between the droplets. This angle is then used to estimate the bilayer tension using $\gamma_b = 2\gamma_m cos\theta_m$. The energy of adhesion -ΔF is defined as the difference between the two monolayer areas the new bilayer area, or $-\Delta F = 2\gamma_m - \gamma_b$.

References

1 Song, L. *et al.* Structure of Staphylococcal alpha -Hemolysin, a Heptameric Transmembrane Pore. *Science* **274**, 1859-1865, doi:10.1126/science.274.5294.1859 (1996).

2 Hwang, W. L., Holden, M. A., White, S. & Bayley, H. Electrical Behavior of Droplet Interface Bilayer Networks: Experimental Analysis and Modeling. *Journal of the American Chemical Society* **129**, 11854-11864, doi:10.1021/ja074071a (2007).

3 Makhoul-Mansour, M., Challita, E. J. & Freeman, E. C. in *ASME 2017 Conference on Smart Materials, Adaptive Structures and Intelligent Systems.* V001T006A009-V001T006A009 (American Society of Mechanical Engineers).

4 Challita, E. J., Makhoul-Mansour, M. M. & Freeman, E. C. Reconfiguring droplet interface bilayer networks through sacrificial membranes. *Biomicrofluidics* **12**, 034112 (2018).

5 Taylor, G. J., Venkatesan, G. A., Collier, C. P. & Sarles, S. A. Direct in situ measurement of specific capacitance, monolayer tension, and bilayer tension in a droplet interface bilayer. *Soft Matter* **11**, 7592-7605, doi:10.1039/c5sm01005e (2015).

6 Challita, E. J., Najem, J. S., Monroe, R., Leo, D. J. & Freeman, E. C. Encapsulating Networks of Droplet Interface Bilayers in a Thermoreversible Organogel. *Scientific reports* **8**, 6494-6505, doi:10.1038/s41598-018-24720-5 (2018).

7 El-Beyrouthy, J., Makhoul-Mansour, M. M., Taylor, G., Sarles, S. A. & Freeman, E. C. A new approach for investigating the response of lipid membranes to electrocompression by coupling droplet mechanics and membrane biophysics. *Journal of the Royal Society Interface* **16**, 20190652 (2019).

8 Makhoul-Mansour, M. *et al.* Ferrofluid-Based Droplet Interface Bilayer Networks. *Langmuir* **33**, 13000-13007, doi:10.1021/acs.langmuir.7b03055 (2017).

9 Makhoul-Mansour, M. M., El-Beyrouthy, J. B., Mumme, H. L. & Freeman, E. C. Photopolymerized microdomains in both lipid leaflets establish diffusive transport pathways across biomimetic membranes. *Soft Matter* **15**, 8718-8727, doi:10.1039/c9sm01658a (2019).

10 Berry, J. D., Neeson, M. J., Dagastine, R. R., Chan, D. Y. & Tabor, R. F. Measurement of surface and interfacial tension using pendant drop tensiometry. *J Colloid Interf Sci* **454**, 226-237, doi:10.1016/j.jcis.2015.05.012 (2015).
