# Supplementary material for: Enhancing membrane-based soft materials with magnetic reconfiguration events
Source: Sci Rep. 2022 Feb 1;12:1703. doi: 10.1038/s41598-022-05501-7 (PMC8807651; doi:10.1038/s41598-022-05501-7)
Supplement: Supplementary file 2 — Supplementary Legends. [file 41598_2022_5501_MOESM2_ESM.docx]

**Video S1:**

An extrusion mechanism is driven via the application of magnetic force fields to a hybrid DIB structure containing ferrofluid and water microdroplets (600 μm in diameter each). For ease in visualization, the playback speed is increased by a factor of 4.

**Video S2:**

A double extrusion/folding mechanism is driven via the application of magnetic force fields to a hybrid DIB structure containing ferrofluid and water microdroplets (600 μm in diameter each). For ease in visualization, the playback speed is increased by a factor of 4.

**Video S3:**

A folding mechanism is driven via the application of magnetic force fields to a hybrid DIB structure containing ferrofluid and water microdroplets (600 μm in diameter each). For ease in visualization, the playback speed is increased by a factor of 4.

**Video S4:**

A neighbor swap (T1) mechanism is driven via the application of magnetic force fields to a hybrid DIB structure containing ferrofluid and water microdroplets (600 μm in diameter each). For ease in visualization, the playback speed is increased by a factor of 4.
